# Exploring pandemic-related health literacy among adolescents in Germany: a focus group study

**DOI:** 10.1186/s13690-022-00937-9

**Published:** 2022-08-05

**Authors:** Anne-Kathrin Mareike Loer, Olga Maria Domanska, Christiane Stock, Susanne Jordan

**Affiliations:** 1 Department of Epidemiology and Health Monitoring, Robert Koch Institute, General-Pape-Str. 62-66, 12101 Berlin, Germany; 2 Institute of Health and Nursing Science, Charité - Universitätsmedizin Berlin, Corporate Member of Freie Universität Berlin and Humboldt Universität zu Berlin, Augustenburger Platz 1, 13353 Berlin, Germany; 3 Unit for Health Promotion Research, University of Southern Denmark, Dengevej 14, 6705 Esbjerg, Denmark

**Keywords:** Health literacy, COVID-19, Pandemic, Adolescents, Qualitative research, Online focus groups

## Abstract

**Background:**

Health literacy enables people to cope efficiently with health threats, such as the COVID-19 pandemic. However, little is known about health literacy among adolescents in general and especially in the context of pandemics. This study aimed to explore pandemic-related health literacy among adolescents by addressing cognitive, behavioral, conative, and affective components of the multidimensional health literacy construct.

**Methods:**

Four online focus groups with 24 adolescents aged 13-17 years from four German federal states were conducted during the COVID-19 pandemic in May and June 2021. Data were analyzed using qualitative content analysis.

**Results:**

Regarding the cognitive and behavioral components of pandemic-related health literacy, adolescents reported to use a broad range of traditional and digital media and personal information sources. The adolescents considered pandemic-related information to be good and easy to understand, when the information is presented in a concise and structured manner. The participants stated difficulties in finding, understanding, and evaluating pandemic-related information regarding particular protective measures. The adolescents described themselves to be critical when evaluating pandemic-related information and reported a high level of adherence to protective measures. Regarding the conative and affective components of health literacy, the adolescents explained that their wish to protect their loved ones from getting infected was the predominant motive for adherence to protective measures. They were convinced that people of their age play a role in pandemic containment. The adolescents reported sometimes making exceptions from adhering to protective measures to cope with negative feelings they experienced during the pandemic.

**Conclusions:**

This study provides insights on how measures to improve pandemic-related health literacy among adolescents may be tailored to their needs. Prompt, concise, structured, and comprehensible preparation and communication of pandemic-related information in addition to educational efforts to strengthen health-related cognitive skills and critical health literacy may be supportive to reduce barriers in finding, understanding, and evaluating pandemic-related content.

**Supplementary Information:**

The online version contains supplementary material available at 10.1186/s13690-022-00937-9.

## Background

The coronavirus disease 2019 (COVID-19) pandemic has had a severe impact on the lives of adolescents [1–3]. They are in a particularly vulnerable phase of life characterized by coping with specific developmental tasks [4]—for example, achieving increased independence from parents, building peer and partner relationships, and developing an autonomous lifestyle also with regard to health behavior [5]. Managing these developmental tasks under pandemic conditions has become a challenge for adolescents: In the context of the pandemic and the implemented protective measures, adolescents had to understand the responsibility of individuals in containing the pandemic by following different protective measures and to decide whether to disregard them in favor of living their own personal desires and needs, or to adhere to them in favor of protecting collective health [6]. Besides these individual factors, adolescents had to deal with societal and environmental factors, such as pandemic-related information overflow, misleading information, and uncertainty of scientific knowledge regarding whether the implemented protective measures were appropriate [6, 7]. Individual health literacy constitutes a core competence to handle these challenges and responsibilities in infection disease prevention [8].

A widely accepted definition by Sørensen et al. [9] describes health literacy as a multidimensional construct and defines generic health literacy as skills and abilities, knowledge, and motivation to find, understand, evaluate, and apply health information for health-related decisions. In the context of the COVID-19 pandemic, pandemic-related health literacy focuses on finding, understanding, and evaluating information on severe acute respiratory syndrome coronavirus type 2 (SARS-CoV-2), the illness COVID-19, and different protective measures, resulting in implementation of the measures in one’s own actions [10]. The definition by Sørensen et al. [9] was developed with respect to adults and does not consider particular characteristics, vulnerabilities, and social contexts in adolescence that are relevant for health literacy [11, 12]. However, Bröder et al. [13, 14] identified specific characteristics of health literacy among children and adolescents, which they refer to as, for example, disease patterns and health perspectives, democracy (active citizenship and participation), and digitalization. Regarding these three characteristics, they state that the provision of health-related information should be oriented toward the health perception of children and adolescents and toward the relevance and meaningfulness of information for them, considering that young people also receive health information from digital media [13, 14]. The authors also advocate listening to and empowering young people to engage with their own health and that of others in an empowered and ethically responsible way [13, 14]. They classified specific categories for young peoples’ health literacy: Cognitive, behavioral, affective and conative components [11]. Cognitive components include mental abilities and actions that are needed to process information, such as understanding or evaluation [11]. Behavioral components refer to concrete actions of individuals, such as seeking information [11]. Affective and conative components are highly interrelated. Affective components refer to experiencing feelings or emotions. Conative components describe personality traits and mental stages that guide health-related action; these include, for example, self-control and self-regulation, which essentially influence how people deal with situations they experience as unpleasant, but also motivation to take responsibility for health-related issues [11]. In relation to the COVID-19 pandemic, finding, understanding, evaluating, and applying health information can be assigned to the *cognitive and behavioral components* [11]. Adolescents’ motivation to adhere to protective measures, their attitudes toward the role of adolescents in limiting the spread of the virus, their experiences of the pandemic, and protective measures on COVID-19 refer to the *affective and conative components*. Accordingly, to explore adolescents’ health literacy during the COVID-19 pandemic, the *cognitive*, *behavioral*, *conative*, *and affective components* of adolescents’ health literacy should be considered.

Despite the great importance of health literacy as a core competence enabling one to cope with health threats such as the COVID-19 pandemic [8], there is limited evidence about health literacy among adolescents in general [15, 16], but especially in the context of pandemic situations. The published studies regarding adolescents and the COVID-19 pandemic have investigated specific aspects of health literacy, such as pandemic-related information sources [17, 18] and knowledge about COVID-19 (*cognitive components*) [17], or social responsibility as a factor associated with pandemic-related health behavior—for example, disinfecting and news monitoring, and motivation to engage in social distancing (*conative components*) [19–21]—or affective experiences of the COVID-19 pandemic (*affective component*) [22, 23]. A few studies have focused on specific areas in which people use health literacy for health-related decisions (specific health literacy) [24, 25], such as mental health literacy [26] or eHealth literacy [27], but the authors did not address health literacy in its entity (generic health literacy). Two studies by Riiser et al. [28, 29], one quantitative and one qualitative, examined several components of generic health literacy among Norwegian adolescents during the COVID-19 pandemic in the year 2020. In the quantitative study, adolescents most frequently mentioned television and family as the main sources of pandemic-related information and the majority of the participants showed adherence to the guideline on protective measures. The study also found associations between health literacy and handwashing knowledge and behavior as well as application of social distancing [28]. In the qualitative study, based on focus groups, adolescents mentioned traditional media as another main information source. In general, adolescents were well informed about protective measures and able to find reliable pandemic-related information. They hardly strived to follow protective measures motivated by solidarity toward people vulnerable to COVID-19, and adherence to social restriction measures diminished their quality of life [29]. To the knowledge of the researchers, the qualitative study by Riiser et al. [29] is the only study that has examined in depth several components of generic health literacy with a special focus on pandemic events among adolescents; evidence regarding this topic in Germany is lacking.

Research on adolescent health behavior during a pandemic can lead to an understanding of adolescents’ needs. The results can then inform the development of interventions that foster adolescents’ adherence to pandemic-related protective measures [30]. Research on adolescent health literacy can also contribute to this effort. During the COVID-19 pandemic, the voices of young people have not been sufficiently heard, which has often resulted in frustrations [22] or the feeling of being overlooked in decision-making processes, for example because school principals and adults have believed they could better assess the needs of adolescents [31]. Studies, respecting adolescents perceptions and voices regarding health literacy and pandemic events, are needed. These studies could reveal the needs of young people in this context. Findings could provide information on how to design and deliver pandemic-related information and how to best tailor protective measures to ensure the acceptance and adherence of adolescents.

This qualitative study aimed to explore the pandemic-related health literacy among 13-17-year-old adolescents, living in Germany, and to gain new insights into the multidimensionality of the pandemic-related health literacy construct by addressing its *cognitive*, *behavioral*, *conative*, *and affective components*. The results obtained by following this methodology appear suitable for understanding the relevance of health literacy during the COVID-19 pandemic and similar infectious events in a field that has been under-researched [32]. Further, to generate implications for practice by considering adolescents’ needs, the researchers investigated how adolescents’ pandemic-related health literacy may be improved.

Specifically, following aspects were examined: *Cognitive and behavioral components* of health literacy were studied by discussing with adolescents about finding, understanding, evaluating, and applying pandemic-related health information regarding SARS-CoV-2, the infectious disease COVID-19, and the protective measures. *Conative and affective components* of health literacy were addressed by talking about adolescents’ motivation to adhere to protective measures (reducing contacts, keeping physical distance, and wearing a face mask), their attitudes toward their role in slowing down the spread of coronavirus, and their experiences of the pandemic. Additionally, to generate implications for practice orientated toward adolescents’ needs, advice from adolescents was sought on how they would like to be informed about pandemic-related protective measures and risks and what would make it easier for them to adhere to protective measures.

## Methods

### Background information on protective measures during different phases of the pandemic in Germany to illustrate the study context

In spring 2020, schools were successively closed throughout Germany and a nationwide lockdown was introduced. Also, a nationwide obligation to wear a face mask was adopted. After a reduction of protective measures in summer, a partial lockdown followed in November 2020, lasting two and a half months [33]. At times of lockdowns, extensive contact restrictions were adopted, for example, in December 2020, private meetings with friends and relatives were continuously limited to one’s own and one other household [34]. Mid-December 2020, schools were closed and partially re-opened in March 2021, depending on the federal states with the consequence that millions of young people had to gain digital school lessons at home [35].

### Study design

For this study, four focus groups with adolescents were conducted. Focus groups allow gaining knowledge and performing an in-depth exploration about perceptions, interpretations, and value judgements on certain topics that are characteristic for a group [36]. In accordance with the outlined research interest, the researchers chose this qualitative method to capture individual and collective perspectives of adolescents regarding their pandemic-related health literacy, experiences, motivation, and attitudes toward SARS-CoV-2, the infectious disease COVID-19, and protective measures. Four focus groups were carried out to cover at least 80% of expected data saturation [37]. The reporting of this study follows the “Consolidated criteria for reporting qualitative research (COREQ): a 32-item checklist for interviews and focus groups” [38].

This study entitled “COVID Pandemic-related Health Literacy Among Adolescents” (COVID-GeKoJu) is a sub-study of the project “Measurement of Health Literacy Among Adolescents” – Part 2 (MOHLAA 2) of the Robert Koch Institute (RKI), which is embedded in the German Health Literacy in Childhood and Adolescence (HLCA) Consortium.

### Participant sampling procedure

In the study design, the researchers intended to generate a purposive sample including the following eligibility criteria: Age (13-15 years old, 16-17 years old), sex (female, male), and attending any type of school. To reach adolescents with diverse school backgrounds, youth and sport clubs in two federal states, Berlin and Brandenburg, were asked to hand out study materials to interested adolescents. These federal states were chosen at a time, where personal visits of the research team to youth and sport clubs were planned for recruitment in order to reduce the travel time. Recruitment started in mid-February 2021, and after 2 months the number of registrations received from adolescents was very low. Based on the feedback from some youth and sport clubs, that they had little or no contact with adolescents due to the protective measures in force at the time of the recruitment in Germany, the sampling procedure was extended to convenience sampling. Adolescents were now also reached through the researchers’ friends, acquaintances, and colleagues, and thus expanded to the federal states Berlin, Brandenburg, Lower Saxony, and North Rhine-Westphalia. As the researchers allowed friends to register together for study participation, the participants were partly know to each other.

### Data collection

**Fig. 1:**
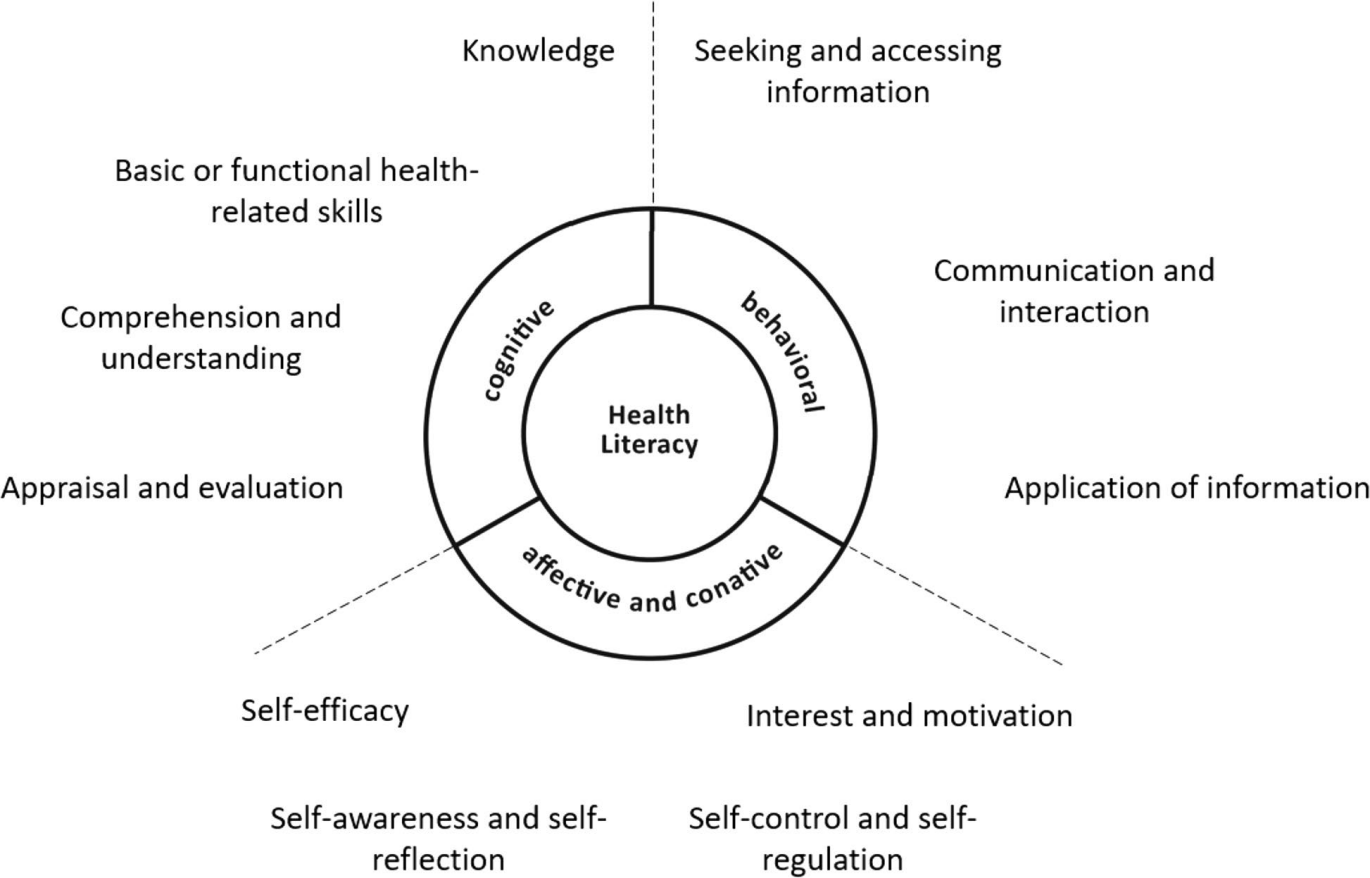
Conceptual framework: Cognitive, behavioral, conative, and affective components of health literacy, adapted from Bröder et al. (2017) [11]

The four focus groups were conducted between May 7 and June 14, 2021, at the end of the third wave of the COVID-19 pandemic in Germany [39]. Information regarding the sample characteristics and focus group composition are presented in Table 1. During this time, learning modalities in schools were characterized by a hybrid of in-person and remote learning, or full remote learning. Due to the dynamic situation of the COVID-19 pandemic and the unforeseeable protective measures required to contain the pandemic, the focus groups were planned from the beginning to be conducted online using Cisco Webex Events, a video-conferencing software [40]. The average duration of each focus group was 2 hours, including a brief 5-10 minute break. The response options were changed inbetween by using the chat function to activate the adolescents (see Table S1). Before conducting the focus groups, the researchers did not establish a relationship with the participants, and the participants had no knowledge of the researchers and their specific characteristics; although participants were recruited via researchers’ friends, acquaintances, and colleagues, the researchers did not have any contact to the participants before. The focus groups were visual and audio recorded with written permission from the participants and their parents/legal guardians. After participation, the adolescents received a voucher (choice: drugstore or electronic store) for 25 euros of credit Fig. 1.

To conduct and to moderate the focus groups, the researchers developed a semi-structured interview guide (Additional file: Table S1), considering pandemic-related health literacy as a multidimensional construct by addressing its cognitive, behavioral, conative, and affective components. The underlying conceptual framework is shown in Fig. 1.

The interview guide was examined by two experts in the field of qualitative research and two experts in the field of gender research and was pilot tested with two young people (aged 16 and 22 years) in a video-conference. To maintain consistency, all focus groups were moderated by one of the two female researchers (AKML), who holds a M.Sc. Public in Health/Epidemiology. The second researcher, who is a sociologist and holds a Dr. rer. medic. (OMD), organized the technical processes during the online event and took field notes.

### Data analysis

Verbatim transcription of the audio data was carried out by four staff members of the department’s secretariat. The researchers checked all four transcripts for completeness, accuracy, and anonymity. Transcripts were not returned to participants for comments and/or corrections. The chat function text was transferred to Word-files for the analysis. The method of qualitative content analysis according to Kuckartz [41] was applied to identify, conceptualize, and systematically describe content-related aspects of the data material [41, 42]. The researchers chose an inductive-deductive category system because it allows for greater openness to additional aspects that are important to the target group [41, 42]. Deductive codes were developed based on the interview guide. In the initial coding phase, both researchers coded text segments together to gain a common understanding of the codes. Subsequently, each researcher independently coded text passages. The researchers compared their codes regarding their correspondences and entered into a discourse in case of discrepancies. Each of the researchers then coded two focus groups. New inductive codes—for example, difficulties in finding pandemic-related health information or experienced lack of appreciation of society—were defined and discussed, and the complete material was coded again with the differentiated category system. For coding consistency, one of the researchers (AKML) checked the coded text passages of her colleague. After the coding phase, both researchers conducted the content analysis, considering age and gender differences, and discussed the results. The analytical approach enabled answering the research questions and identifying emerging themes. The researchers used the qualitative analysis software MAXQDA® 2020 to manage the data [43].

## Results

A total of 24 adolescents aged 13-17 years from four federal states in Germany (Berlin, Brandenburg, Lower Saxony, North Rhine-Westphalia) took part in this study. Most of the participants attended a grammar school. The sample characteristics are presented in Table 1.

**Table 1:** Sample characteristics of the study participants “COVID Pandemic-related Health Literacy Among Adolescents” (COVID-GeKoJu) (*n* = 24), 2021

Focus group	Number of participants	Age in years	Sex	Type of school		Federal states represented
				Grammar school visit	Non-grammar school visit	
1	6	13-15	Male	6	0	Berlin, Brandenburg, Lower Saxony
2	5	16-17	Male	5	0	Berlin, Brandenburg, Lower Saxony, North Rhine-Westphalia
3	6	13-15	Female	6	0	Berlin, Brandenburg, Lower Saxony
4	7	16-17	Female	5	2	Berlin, Brandenburg, Lower Saxony

### Cognitive and behavioral components of pandemic-related health literacy

#### Seeking relevant information concerning daily life

The adolescents reported that they most frequently searched for pandemic-related information in the Internet, especially using the search engine Google. On the Internet, they preferred official “Corona-related websites” of the federal state, county, or city in which they reside. Besides the Internet, some adolescents also occasionally sought information in traditional media, such as TV news, but also local radio stations or newspapers. They also consulted others, mainly parents, but also friends, teachers, and acquaintances (e.g. other people of their age or police officers from the family´s circle of acquaintances), as information sources. The situational accessibility of sources determined whether young people used the Internet or their parents first. Adolescents, though, did not just actively seek out information: Many stated that information regarding protective measures and their changes was often passed on to them, mostly by school (e.g., via their school’s website), but also by people in everyday situations (e.g., parents), or social media (e.g., posts on Instagram): “[...] *and then when there’s a relaxation* [of protective measures]*, then you also notice that, um, just at school then they talk about it or also at home or something.*” (ID18, focus group 1)

The need to seek information differed and was less influenced by age or gender. Some participants reported that their information-seeking behavior decreased considerably during the course of the pandemic, for example, due to difficulties in finding information, and only instantly needed information was subsequently searched. At the time of focus groups, they most frequently sought information about protective measures that were important for them directly in their everyday life: “*Um, well, I think it’s somehow quite important to know, um, how many people you’re allowed to meet or when stores open or something like that. But I don’t necessarily need to know how high the incidence is in other countries or anything like that. It’s more about things that affect me directly, and I’d like to be informed about them.*” (ID42, focus group 4)

#### Lack of concisely communicated information

When asked which pandemic-related information is good and easy to understand, many of the adolescents answered that this was the case when information is presented in a concise and structured manner: “*So, the information should already be concise and formulated in not too much detail so that it becomes uninteresting; for us as a younger population, lengthy, formulated information is completely unnecessary and superfluous.*” (ID18, focus group 1)

In contrast, information was described several times as not being good if the adolescents had to read through several pages to find the content relevant to them. As a negative example, multi-page ministerial letters forwarded by school principals were mentioned as being too long and also too difficult to understand. Therefore, several participants advocated for the use of bullet points with keywords in bold or short summaries. The prompt availability of information, especially about changes in regulations on protective measures, played an important role in particular for 16-17-year-old boys: “*Uh, so for me, I think good information is also information that you can get quickly and you don’t have to search for a long time.*” (ID16, focus group 2)

Several adolescents experienced difficulties in finding and understanding information regarding protective measures, especially for contact reduction (“social” distancing). The perceived difficulties in finding and understanding information varied in different periods of the pandemic. In times of a partial lockdown, there was less confusion due to clearly set information on the duration of measures and rules. At the time of the focus groups in the beginning of summer 2021, it was perceived as difficult to find out which rules were currently in place. They reported that others, including adults around them, did not understand the rules. Girls in particular communicated several times difficulties in finding information and a lack of clarity about existing regulations on protective measures: “*But if you really want to find out how many people you can meet, it’s not that easy. At the moment, no one in the region knows exactly what’s going on here, also with the low incidences, which is just confusing.*” (ID17, focus group 4)

The participants mentioned that regulations communicated on news channels or via press conferences were difficult to comprehend. Several times, they also reported uncertainties regarding current protective measures because of constant changes in the regulations. The existing divergence of regulations at the regional (county) and national levels, as well as across the German federal states, also confused the adolescents: “*Uh. Yes, I also think that things have improved at the moment, but it was really partly the case that I didn’t know, um: What’s going on right now? So, can I now also meet with two people or only with one? Or, what about the masks?* […] *Uh, that always changed pretty quickly, and I didn’t always know exactly where to look.*” (ID23, focus group 3)

The adolescents stated different strategies as solutions for how to deal with uncertainties. For example, considering the great pace of changing regulations, one older girl stopped informing herself daily at a certain point and only researched information on protective measures when needed. Another girl reported that she simply terminated the search process and decided to act without knowing the exact measures when it took too much effort to find the needed information.

#### Trustworthiness of information from parents, schools, and established media

In all focus groups, it became apparent that adolescents proceeded similarly when appraising pandemic-related information. For example, they compared different sources of information with each other to see if the information matched. To compare information, they also consulted interpersonal sources such as their parents, teachers, or friends: “*So um, I would definitely first, I think, ask my parents, um, if they have somehow already heard something about it and if they don’t know anything either, then I would, I think, because we have a class chat with teachers, I would then ask whether the teachers somehow know whether that’s true or not and, yes.*” (ID32, focus group 4)

To appraise the seriousness and credibility of pandemic-related information, the adolescents named several criteria whereby the distinction between a serious information and a serious information source could not be clearly disentangled in the adolescents’ statements. In all the focus groups, the trustworthiness of an information source, such as media, institutions, or individual people, was the main criterion for appraising the credibility and seriousness of information: “*But I also rather think that it’s not at all about the information itself that's written in there, but more about the format, so for example, from what source, for example, if that’s the principal, then of course you’re more likely to believe that than if that’s some website that you’ve never heard of. Um. So, I find that the source or the format, uh, is even more important than what is actually really written in there, because I personally cannot judge.*” (ID19, focus group 1)

Schools and parents, but also established and well-known sources (e.g., traditional media: public-service channels or newspapers; digital media: specific podcasts (e.g. a podcast of the news program „Tagesschau“) on the audio streaming service Spotify), were considered as serious or trustworthy sources of information. Information provided by parents was usually fully trusted by younger participants, but also checked by older participants. Compared with information provided by parents or school, information provided by other people such as classmates was more often checked for trustworthiness. In general, younger boys were less likely to report having been confronted with pandemic-related information that they did not know whether it was true. In contrast, several older boys affirmed that they had experienced uncertainties evaluating information. Besides trustworthiness, the participants mentioned additional evaluation criteria, such as plausibility and logic of information, or information sources with listed references, and a serious appearance.

#### Adherence to the protective measures and the role of exceptions

Most adolescents reported following the protective measures, reducing contacts, maintaining physical distances, and wearing a face mask, but also making exceptions. Regarding contact reduction, adolescents reported some exceptions, at different situations—for example, at birthdays (more households than allowed came together), at Halloween, or after passing the school-leaving examination. Regarding physical distancing, adolescents made exceptions—for example, because they were not used to maintaining distance in contact with close people and therefore quickly fell back into habitual patterns or because they simply forgot to maintain physical distance. Regarding facemasks, adolescents communicated that they removed facemasks at school—for example, when experiencing discomfort of not getting enough oxygen while spending several hours in a room with a facemask.

When talking about exceptions, several participants referred to having made them under certain circumstances. Two girls reported having built up their peer group appropriately to fit the contact restrictions, while one of the girls reported that in her group of friends, they did not pay attention to distance during meetings. Furthermore, small group meetings (four to five people), which were not allowed at that time, took place, but outside and with physical distancing and negative SARS-CoV-2 test results. Having a negative test result was mentioned several times when the adolescents talked about exceptions; for example, it was the motive for no longer keeping sufficient physical distance or no longer wearing facemasks. According to a younger girl, who was part of a small peer group, the testing facilities made it possible not to exclude a person in the group from peer meetings: “*Yes, so now there were also not so many, so I think at most there were* [...] *three or four people, so there were also not so many and it was just still not allowed, so to speak, but we then met when we tested ourselves, when we then knew that we were all negative.*” (ID32, focus group 4)

Following the measures was harder or easier for them depending on the context. For example, adherence was easier in the summer under less severe measures as well as in the winter due to bad weather conditions, in public spaces, and after introduction of SARS-CoV-2 test facilities. In contrast, following the measures was harder at school, but also due to contradictions in the protective measures (physical education classes were allowed, but private meetings after school with the same people from class were not). One boy described that following the rules was particularly difficult when he knew that other friends were meeting, even in larger groups.

### Conative and affective components of pandemic-related health literacy

#### Care for others as the main motivation for adherence to protective measures

The primary concern for adolescents’ adherence to protective measures was the protection of others, especially family members (parents, grandparents, and elderly relatives), but also other people (schoolmates, at-risk patients, and elderly neighbors). By following the measures, they wanted to avoid spreading the virus through their own behavior, which could put other people at risk. Another important motive for adhering to the measures was the aspiration that the pandemic would be overcome earlier as a result of their cooperation: “*And then maybe this thought that, um, if you stick to it, there’s an end in sight, so if everyone really sticks to it and does their best, uh, contributes their best. Then you can also see the light at the end of the tunnel.*” (ID22, focus group 2). Only a few adolescents mentioned other motives for adherence to protective measures, such as to avoid one’s own serious illness, fear of hospitalization or post-COVID conditions, or fear of isolation, or being the reason for quarantine of their classmates if they got infected.

#### The responsibility of adolescents as compared with adults

Among the adolescents, there was a consensus that people of their age play a role in limiting the spread of the virus. They expressed awareness for their responsibility in terms of pandemic containment, because young people could also spread SARS-CoV-2. Therefore, they widely agreed that they have the same responsibility as other citizens: “*Uh, yes, I think we all play the same role or have the same responsibility for it, even the younger ones, I think. Because everyone can have that, uh, everyone can carry it with them and also infect others and, uh, yes, good.*” (ID16, focus group 2). One girl emphasized the high burden of responsibility that adolescents have to carry during the pandemic. While they are in a phase of life in which they want to explore new things, they now see themselves burdened with the responsibility of preventing close people such as relatives from dying due to infection: “*Yes, and I also think it is so extreme that we just have to take this whole responsibility on ourselves, uh, as people who actually still want to discover life a little bit and so and normally should be on the road even more as adults. We are nevertheless responsible for the lives of our relatives or for other people around us.*” *(ID13, focus group 3)*

A recurring topic was the lack of appreciation, support, gratitude, and attention that they experienced for their responsible behavior during the pandemic. According to a girl, for example, adolescents are often poorly portrayed in the public, even though a majority of them are highly committed to containing the pandemic. The participants mentioned specific situations in which adults or older people have behaved as poor role models, resulting in doubts with respect to their own adherence: “*Then there is simply a lack of acceptance and then you start to think about it: Yes, okay, but if those who say: It’s our* [adolescents’] *fault, but don’t stick to it [protective measures] themselves, why should we stick to it?*” (ID27, focus group 3)

#### Forced neglect of close contacts

When asked for motives to non-adherence to social distancing measures, the participants mentioned not seeing grandparents and friends for a long time, and experiencing monotony and mental stress due to the pandemic. In addition, among older boys there were concerns about losing their peer group when not attending gatherings as motives for renouncing contact reduction: “*So then maybe you just make an exception, because you think it’s going to take so long anyway, I don’t want to wait that long. Then I’ll meet with them anyway. Uh, so that I don’t lose them now as friends perhaps or am no longer in there* [peer group]*.*” (ID16, focus group 2) Almost all of the 13-17-year-old adolescents described negative experiences in relation to the ongoing current pandemic situation like being annoyed, frustrated, strained, and demotivated by the prolonged situation due to recurring restrictive measures in the course of partial lockdowns. They also reported fear of additional partial lockdowns and a never-ending pandemic: “*Because in the meantime, at some point in the month of March, it may have happened that one now just always stays at home, that there is never any hope for improvement and that then perhaps the thought comes up, that it won’t get better anyway. Now I’m meeting with people, now, it doesn’t matter anyway, what I’m doing.*” (ID35, focus group 2)

The discussions revealed the strong impact of restriction measures on social contacts, resulting in a lack and loss of contact to family members, but also to friends. For an older boy, adherence to social distancing meant that he felt he was “*left behind*” (ID24, focus group 2) and that he had lost the connection to his peer group, which continued to have gatherings even in larger groups. One boy was frustrated about no longer being invited to gatherings by his friends. He was unsure if they did not ask him any longer to join them because they knew about his adherence to protective measures, or if they did not want to have contact with him any longer. The high impact of social distancing measures on peer groups was described very vividly by a girl: “[…] *but I would say in general that Corona has demanded or forced you to make clear cutbacks and also to neglect friends, you can still feel that. With us, the group has also split up, many friends are now alone and don’t really have anyone, which is also a shame.*” (ID17, focus group 4)

### Direct advice from adolescents

#### Preferences for apps and websites

When asked how they would like to be informed about risks and protective measures in the case of a new pandemic, the participants advocated the use of an app or websites to get information. Possible content should include information about protective measures and changes in regulations for the federal states as well as for specific places of residence and for adolescent-specific environments (e.g., schools). They considered promptness and clarity of information to be highly important. The use of social media as an information channel was only discussed in the focus group of older male adolescents. Arguments for the use of social media were the speed of information transfer and practicability aspects, as young people access social media like Instagram or TikTok several times a day.

#### Increasing the adherence to protective measures

In both focus groups with male participants, the participants mentioned several times that, in their view, the prospect of an improvement in the pandemic situation would make it easier for adolescents to adhere to protective measures. Several boys shared the opinion that measures should be implemented that make it easier for young people to meet other people and to engage in sports activities. They expressed that this concession for the needs of young people could in turn lead to higher motivation to adhere to the protective measures. On the other hand, several girls considered it important that the measures be consistent for all age groups and affect the living conditions to a similar extent, but also that the regulations should be followed across all age groups.

## Discussion

This qualitative study aimed to explore the pandemic-related health literacy among 13-17-year-old adolescents living in Germany by addressing its *cognitive*, *behavioral*, *conative*, *and affective components.* With respect to this research aim, the researchers have gained new insights on the following two aspects.

### Finding, understanding, evaluating, and applying pandemic-related health information (*cognitive and behavioral components*)

Regarding the *cognitive and behavioral components* of pandemic-related health literacy, the researchers found that adolescents used the Internet as their main source of information, but they also consulted people from their direct social environment. They preferred pandemic-related information to be prepared in a concise and structured manner. Difficulties in finding and understanding information were related to information on protective measures. The experienced difficulties were mainly explained by the fact that the measures changed constantly during the course of the pandemic. However, the adolescents stated concrete practices for seeking as well as verifying pandemic-related information. They defined clear criteria for assessing whether information is true or fake; for example, established sources were rated as being trustworthy. The vast majority followed the protective measures, but sometimes they made exceptions.

The results of this study regarding information sources go in line with other studies focusing on adolescents describing their use of newspapers, the Internet, or interpersonal sources for seeking information [17, 18]. As in the focus groups of this study, asking interpersonal sources, especially family, for pandemic-related information was also common among adolescents in the both studies cited. In contrast, no consistent results are yet available for adults: The use of interpersonal information sources among adults ranges from less than a quarter [44] to three quarters [45]. Therefore, it remains open if the use of interpersonal resources for receiving pandemic-related information is specific for adolescents or can also be observed in adults.

The results of this study indicate that difficulties and uncertainties in finding, understanding, and evaluating information differed by age and gender. Girls more frequently reported difficulties in finding and understanding information than boys, and older boys more often communicated they had experienced uncertainties while evaluating information than younger boys. This result is in accordance with a representative cross-sectional study for assessing generic health literacy among German 14-17-year-old adolescents (*n* = 1,235) [25], conducted by the researchers, showing in bivariate analyses that female and older adolescents more often reported they had many or some difficulties in dealing (finding, understanding, evaluating, and applying) with health-related information than male or younger adolescents [25]. The age differences found may be explained by the cognitive development in adolescence, including critical thinking skills. At the beginning of adolescence, young people are already aware that information can be true or false; however, the competence of critical appraisal of several sources of information against each other is acquired throughout adolescence [46]. Therefore, the observed differences in defining problems while evaluating information by age may be attributed to different stages in the cognitive development of adolescents in the sample: Younger adolescents may not yet be used to evaluating information in depth, and therefore do not experience difficulties or uncertainties. Regarding gender, more research is needed to get a deeper understanding of the observed differences in experienced difficulties. For example, it remains open to what extent the responses of girls and boys are influenced by gender stereotypes; for example, boys may tend to perceive themselves as more competent than girls, even though the abilities of boys and girls may not differ *per se*. However, attributing difficulties and uncertainties to sociodemographic attributes such as age and gender solely is insufficient: From the comments of the adolescents, it became clear that difficulties are also caused by external circumstances—for example, due to rapid changes in protective measures.

### Motivation to adhere to protective measures, attitudes toward their role in in slowing down the spread of coronavirus, and their experiences of the pandemic (*conative and affective components*)

The adolescents in the focus groups showed high adherence to protective measures. This is in line with studies from Germany and other countries demonstrating that a large proportion of young people followed the diverse protective measures [17, 19, 28, 47, 48].

The researchers found that protecting others was the main motive for adherence to protective measures. Adolescents knew how infections occurred and how preventive measures could limit the spread of the virus. Based on this pandemic knowledge, adolescents recognized that they, like other population groups, have to adhere to protective measures to fulfill their social responsibility. The adolescents consistently expressed that people of their age play a role in pandemic containment. Oosterhoff and Palmer [20] also found the impact of a perceived responsibility on adherence to protective measures when examining attitudes and psychological factors associated with pandemic-related behavior among adolescents aged 13-18 years. There was a positive association between social responsibility and disinfecting and news-monitoring behavior [20]. Besides social responsibility, care for others was also stated as a motive for adhering to protective measures, a result that is also in line with other studies [21, 49, 50]. In one of these studies, young adults were more likely to engage in preventive behaviors regarding COVID-19 when they wished to protect their family/friends [50]. Therefore, *conative components* of health literacy seem to reinforce adolescents’ adherence to protective measures.

Adherence to the protective measures and several partial lockdowns negatively affected the adolescents’ mental well-being and social life. The adolescents in the focus groups, for example, explicitly reported they had experienced mental stress. Other research has also reported negative experiences of adolescents due to the pandemic [23, 51, 52]. The pandemic exemplified the importance of mental health literacy to strengthen adolescents’ competencies to cope with experienced mental health problems [26]. The results of the focus groups showed that adolescents also used a strategy to deal with stress and unpleasant feelings, which was to make exceptions from following the protective measures. However, the researchers found that adolescents more frequently reported exceptions related to specific developmental tasks—for example, exploring new options and establishing relationships with peers. The pandemic situation has severely limited the possibilities of young people gaining these age-specific experiences. They have had to spend a lot of time in their parental home due to school closures, could only have limited social contact with peers, and had to forego activities such as playing sports in a sports club. In favor of these tasks, they occasionally made exceptions to the measures, such as allowing themselves to meet friends to maintain a connection with their peer group. Therefore, the *affective components* of health literacy—but also coping with developmental tasks—favor exceptions from the protective measures.

The second aim was to generate implications for practice by considering adolescents’ needs. With respect to this aim, the results of this study as revealed the following two implications for practice.

### Provide prompt, concise, structured, and comprehensible information using traditional and digital media and interpersonal communication

The findings showed that adolescents would prefer digital channels via apps or websites to receive information about risks and protective measures in case of a new pandemic. In the focus groups, it became apparent that young people access different sources to find pandemic-related information, such as newspapers, the Internet, or interpersonal sources. Therefore, a diverse range of media channels (traditional media and digital media) should be used to reach them. Especially the use of the Internet (apps, websites, social media) seems to be a very promising approach to reach out to young people. Social media in general plays an important role in adolescents’ life and a majority accesses social media regularly or at least daily [53, 54]. The results of a German longitudinal study showed that around two thirds of 10-17-years-olds used social media daily before the pandemic while almost three quarters did so during the pandemic [53]. Social media was a minor topic in the discussions about finding information. When it came to the question of how adolescents want to be informed about pandemic-related protective measures and risks, only older boys recommended social media such as Instagram or TikTok to get informed. Besides the results of this study, other studies have shown that the use of social media for providing pandemic-related information appears to have a certain potential: Social media seemed to be a promising platform to offer health-related content for adolescents [55], and was also a promising communication tool for health authorities [29]. However, the results indicate that the importance of social media should not be overestimated. Additional research is needed to understand how to use social media effectively to convey pandemic-related information to adolescents.

Of note, all information that is communicated, regardless of the medium and the source, should be clearly targeted to the needs of young people, namely being prompt, concise, structured, and comprehensible. A more straightforward communication of containment measures is required: Regulations must be communicated so that they are easy to find and to understand by adolescents and they address their life circumstances and contexts.

### Implications for the practice of promoting health literacy: The role of schools

The results also revealed the importance of promoting pandemic-related health literacy in adolescence. The insights of the focus groups indicated that health-related cognitive skills and critical health literacy, which are described as more advanced cognitive skills used to evaluate information critically [56], are needed to deal with health information. Schools play a crucial role in developing basic capabilities and skills, such as cognition, that are needed for the critical evaluation of different, maybe even contradictory, information, which is needed for health-literate actions [25, 57]. In turn, these actions are needed for the critical evaluation of different, maybe even contradictory, information. The current pandemic situation provides a window of opportunity, because everyone has to deal with fake news and misinformation [6, 7]. The task of critically evaluating misinformation—but also finding serious information—may catch the adolescents’ attention and interest because it directly affects their daily life, and it can therefore be well addressed in schools. Another important issue is the role of schools as a suitable information source. The researchers found that schools are relevant settings for conveying pandemic-related health information, a finding that is also supported by other studies [17, 18]. The adolescents reported they actively consulted teachers for receiving information, but also receiving unsolicited information from school. In addition, the results show that adolescents trusted information provided by schools. Schools have an advantage as providers of health information because, due to compulsory education, all young people, including those from educationally deprived families, can be reached.

#### Limitations

The findings of this studies need to be discussed by taking some limitations into account. The sampling procedure had to be changed to convenience sampling. Therefore, the sample of adolescents in the focus groups of this study was not composed as it was initially intended. The recruitment of adolescents through researchers’ friends, acquaintances, and colleagues may have reinforced a selection bias. As most of the researchers´ contacts had an academic background, recruitment especially took place within academic communities. This bias is reflected in the sample characteristics of the study population regarding educational background: The sample was highly homogenous in terms of the type of school as all participants apart from one were in grammar school. Evidence shows that people with a higher educational background have higher health literacy levels [58, 59]. Including adolescents visiting other school types than grammar schools may therefore lead to different results. On the other hand, the convenience sampling resulted in broader geographical coverage, with participants from different German federal states. Because the protective measures partly differed in the federal states during the COVID-19 pandemic, the focus groups were thus enriched by a wider range of experiences regarding public communication of COVID-19-related information and a different implementation of protective measures. Regarding data saturation, there was a high degree of overlap in the statements in many of the discussed topics, but there were also outlier topics in specific group discussions. For example, concerns about losing the peer group were discussed in depth only among 16-17-year-old boys. It remains an open question as to whether conducting additional focus groups would have led to additional findings; therefore, the results should not be interpreted as being exhaustive. The focus groups took place at a very specific period of time within a pandemic and research findings specific to the topic are therefore challenging to re-confirm. However, future research regarding pandemic-related health literacy, also in the context of other pandemics, should aim at including adolescents with diverse educational backgrounds to broaden the insights.

## Conclusions

This qualitative study has provided new insights into pandemic-related health literacy among adolescents living in Germany by exploring *cognitive*, *behavioral*, *conative*, *and affective components* of health literacy. The results indicate how pandemic-related health literacy among adolescents may be improved by tailoring the communication of COVID-19-related information to their expressed needs.

A range of media outlets (traditional and digital media and interpersonal resources), especially using the Internet, may be effective to reach adolescents on a broad level. The researchers found a need to reduce barriers in finding, understanding, and evaluating pandemic-related content; prompt, concise, structured, and comprehensible preparation and communication of pandemic-related information as well as educational efforts to strengthen health-related cognitive skills and critical health literacy on the other hand may be supportive to address these issues. Policy makers should be aware of the strong impact of protective measures on adolescents’ affective experiences when considering the implementation of future protective measures. The adolescents’ statements indicate that interventions to maintain adherence to the measures and to improve their life situation in a pandemic should address the following factors: To enable maintenance of social contact with family and friends; to offer possibilities to engage in sports activities; to avoid measures that restrict adolescents more than adults; to give adolescents sincere appreciation and gratitude for their high commitment; and to recognize, to listen to them, and to address their needs by involving adolescents in the development of protective measures.

In conclusion, the findings of this study underline the importance of *cognitive*, *behavioral*, *conative*, *and affective components* of health literacy in dealing with health threats such as the COVID-19 pandemic. Therefore, all these components need to be addressed when developing interventions to promote pandemic-related health literacy.

## Supplementary Information

**Additional file 1.**

## Data Availability

The analyzed data sheet with transcripts is available in the German language from the corresponding author upon reasonable request.

## Abbreviations

COVID-GeKoJu: COVID Pandemic-related Health Literacy Among Adolescents
HLCA Consortium: Health Literacy in Childhood and Adolescence Consortium
MOHLAA-Q: Measurement of Health Literacy Among Adolescents-Questionnaire
MOHLAA 2: Measurement of Health Literacy Among Adolescents - Part 2
RKI: Robert Koch Institute
