# Supplementary material for: Exploring pandemic-related health literacy among adolescents in Germany: a focus group study
Source: Arch Public Health. 2022 Aug 5;80:182. doi: 10.1186/s13690-022-00937-9 (PMC9354333; doi:10.1186/s13690-022-00937-9)
Supplement: Supplementary file 1 — Additional file 1. [file 13690_2022_937_MOESM1_ESM.docx]

| **Table S1.** Semi-structured interview guide (study “COVID Pandemic-related Health Literacy Among Adolescents” (COVID-GeKoJu) 2021: n = 24) | |
| --- | --- |
| **Topic or domain** *(Component of health literacy)* | **Questions** |
| Introduction into the theme *(Affective)* | In November 2020, the so-called “partial lockdown” was implemented in Germany.  How was that like for you? How did you feel about it? |
| Finding pandemic-related health information regarding the coronavirus SARS-CoV-2, the infectious disease COVID-19, and the protective measures *(Behavioral)* | If you want to know if you can meet with four friends in the park this weekend on Saturday and during daytime, how would you proceed?  How and where do you seek this information? |
| Understanding pandemic-related health information regarding the coronavirus SARS-CoV-2, the infectious disease COVID-19, and the protective measures *(Cognitive)* | How should good information look like?  What kind of information do you think was good?  What kind of information was easy to understand? |
| Evaluating pandemic-related health information regarding the coronavirus SARS-CoV-2, the infectious disease COVID-19, and the protective measures *(Cognitive)* | How do you evaluate whether the information you find is correct? |
| Applying pandemic-related health information regarding the coronavirus SARS-CoV-2, the infectious disease COVID-19, and the protective measures *(Behavioral)* | When was it easy for you to adhere to the measures?  When was it difficult or impossible?  Did you make exceptions in certain situations? |
| Experiences of the pandemic *(Affective)* | Please write in the chat the very first word that comes to your mind about how you felt about the protective measure to reduce contacts/keep physical distance/wear a face mask when it was introduced.  And now the very first word that comes to your mind about how you feel about the protective measure to reduce contacts/keep physical distance/wear a face mask after so many months. |
| Motivation to adhere to protective measures (reducing contacts, keeping physical distance, and wearing a face mask) *(Conative)* | What motivates you to (not) adhere to the protective measures or to make exceptions sometimes? |
| Attitudes toward adolescents´ role in slowing down the spread of coronavirus *(Conative)* | What role do you think adolescents play in slowing down the spread of the coronavirus? |
| Implications for practice orientated toward adolescents’ needs | Imagine that in half a year another virus will occur, which is very easily transmitted from person to person. How would you like to be informed about risks and protective measures? |
| Implications for practice orientated toward adolescents’ needs | Which suggestions or advice do you have that would make it easier for young people to adhere to protective measures? |
